# P64 Accurate incidence and prevalence estimates are important for families of children with juvenile idiopathic arthritis

**DOI:** 10.1093/rap/rkac067.064

**Published:** 2022-09-28

**Authors:** Richard Beesley

**Affiliations:** Juvenile Arthritis Research, Tonbridge, United Kingdom

## Abstract

**Introduction/Background:**

Juvenile Idiopathic Arthritis (JIA) is a heterogenous group of autoimmune disorders characterised by chronic joint inflammation, affecting children and young people (CYP) under the age of 16. Recent analysis in the UK [1] has provided an update to the estimated incidence and prevalence of JIA.

Whilst the policy and health-care benefits of accurate incidence and prevalence rates are understood, this patient- and parent-led qualitative project sought views from parents of CYP with JIA to understand whether knowing accurate estimates of incidence and prevalence are important to them.

**Description/Method:**

Parents of CYP with JIA, all members of a small online group on social media, were asked whether knowing accurate rates of JIA were important to them, and why. Responses were collated and summarized.

**Discussion/Results:**

Whilst a few respondents felt it did not matter to them what the overall rates of JIA were, focusing instead on their own personal experience with the condition, the majority did want to know and gave consistent reasoning.

They advised that, when your child is diagnosed with JIA, it is a very isolating experience. Most people have never heard of the condition before, and being told your child has arthritis is a frightening time (especially if you then become aware of some of the significant long-term effects, such as potential continuation of disease into adulthood and possible joint damage, and the side effects of treatment, and social impacts of the disease [2, 3]. Whilst direct support is available to families affected by JIA (www.jarproject.org/hope), understanding prevalence rates can help reduce mental health burdens on patients and parents and reduce feelings of isolation.

Families also advised that they want to know numbers of children with JIA so we collectively can get a better understanding of the cause, the trends, and the possible reasons behind changes. Knowing accurately the numbers of children with JIA can help to raise awareness; current lack of awareness in society and primary care contributes to delays in diagnosis and potentially worse clinical outcomes [4]. In addition they reported the need for appropriate resourcing of paediatric rheumatology and support services, and the political and financial discussions that must take place to enable that to happen need to be supported by evidence.

**Key learning points/Conclusion:**

Overall, parents do want to know how many other children have JIA. It takes some of the isolation and anxiety away to know you are not alone. It matters because knowing there are other parents in the same situation as you can help reduce anxiety and loneliness. It matters, because knowing there are researchers focussing on JIA helps remind you that work is underway to better understand JIA, its causes, and improved treatments which will eventually lead to improved care for children with the condition. It matters, because it can help target resources appropriately. It matters, because raising awareness is more effective if we know how many people are affected by a chronic condition. And it matters, because as improved treatments are developed we want to be able to see the number of children categorised as having active JIA to reduce and health outcomes for each child to improve.

**References:**

1. Costello R, McDonagh J, Hyrich K, Humphreys J. Incidence and prevalence of Juvenile Idiopathic Arthritis in the United Kingdom 2000-2018: results from the Clinical Practice Research Datalink, Rheumatology, 2021; keab714, https://doi.org/10.1093/rheumatology/keab714

2. Minden K, Niewerth M, Listing J, Biedermann T, Bollow M, Schöntube M, et al. Long-term outcome in patients with juvenile idiopathic arthritis. Arthritis & Rheumatism. 2002;46(9):2392-401.

3. Moorthy LN, Peterson MG, Hassett AL, Lehman TJ. Burden of childhood-onset arthritis. Pediatric Rheumatology. 2010;8(1):20.

4. Rapley T, May C, Smith N, Foster HE. ‘Snakes & Ladders': factors influencing access to appropriate care for children and young people with suspected juvenile idiopathic arthritis - a qualitative study. Pediatr Rheumatol Online J. 2021;19(1):43.

